# A New Polar Transfer Alignment Algorithm with the Aid of a Star Sensor and Based on an Adaptive Unscented Kalman Filter

**DOI:** 10.3390/s17102417

**Published:** 2017-10-23

**Authors:** Jianhua Cheng, Tongda Wang, Lu Wang, Zhenmin Wang

**Affiliations:** College of Automation, Harbin Engineering University, Harbin 150001, China; chengjianhua@hrbeu.edu.cn (J.C.); heuwanglu@hrbeu.edu.cn (L.W.); 2012042133@hrbeu.edu.cn (Z.W.)

**Keywords:** transfer alignment, star sensor, SINS, polar region, lever-arm effect, large misalignment angle, AUKF

## Abstract

Because of the harsh polar environment, the master strapdown inertial navigation system (SINS) has low accuracy and the system model information becomes abnormal. In this case, existing polar transfer alignment (TA) algorithms which use the measurement information provided by master SINS would lose their effectiveness. In this paper, a new polar TA algorithm with the aid of a star sensor and based on an adaptive unscented Kalman filter (AUKF) is proposed to deal with the problems. Since the measurement information provided by master SINS is inaccurate, the accurate information provided by the star sensor is chosen as the measurement. With the compensation of lever-arm effect and the model of star sensor, the nonlinear navigation equations are derived. Combined with the attitude matching method, the filter models for polar TA are designed. An AUKF is introduced to solve the abnormal information of system model. Then, the AUKF is used to estimate the states of TA. Results have demonstrated that the performance of the new polar TA algorithm is better than the state-of-the-art polar TA algorithms. Therefore, the new polar TA algorithm proposed in this paper is effectively to ensure and improve the accuracy of TA in the harsh polar environment.

## 1. Introduction

As various countries pay more attention to the polar region, an increasing number of military and civil activities are occurring in the polar region. Among the different kinds of polar navigation techniques, the strapdown inertial navigation system (SINS) can perform best in the polar region [1,2,3]. In order to achieve a high accuracy of initial alignment in a short time, transfer alignment (TA) of SINS is the optimal choice for moving base alignment [4,5]. However, due to the meridian convergence, north-oriented SINS would suffer some problems in the polar region, such as overflowing calculation and increasing errors [6,7,8]. Similarly, the state-of-the-art TA algorithm based on the north-oriented SINS would have the same problems.

A grid frame and the grid inertial navigation mechanization proposed in [9]. By replacing the traditional line of north orientation by the Greenwich meridian, the grid frame can solve the navigation problems caused by meridian convergence. Based on the grid frame, a linear polar TA algorithm is present in [10], which can be effective to ensure the TA accuracy in the polar region. In the case of a large azimuth misalignment, angle a nonlinear polar TA algorithm is proposed in [11]. However, due to the different installation locations of master and slave SINSs, lever-arm effect exists and would decrease the accuracy of TA. To eliminate the influence of lever-arm effect, a polar TA algorithm with the compensation of lever-arm effect is proposed in [12]. But the compensation algorithm is derived under the small-misalignment-angle assumption. Therefore, it is necessary to derive a lever-arm effect compensation algorithm in the case of a large azimuth misalignment angle. Otherwise, the polar TA algorithm proposed in [8] still use unscented Kalman filter (UKF) to estimate the misalignment angles. However, due to the harsh polar region environment, such as strong winds and big waves, the ship swing would be violent. Once the length of ship is less than the surge wave length, the violent ship pitching would leave propellers idle above water, which would cause an excessive vibration of the ship’s hull. Influenced by the vibration and temperature variation, the error characteristics of inertial measurement unit (IMU) and external ancillary equipment would change. In this condition, the statistical characteristic of noise would be uncertain, which would decrease the accuracy of UKF and even cause a failure of UKF. Thus, the effectiveness of UKF would be influenced and reduced by the uncertain statistical characteristic of noise. An adaptive UKF (AUKF) can adaptively adjust the state information by using measurement information and residual error [13,14]. Thus, an AUKF is introduced to adjust the harsh polar environment.

The polar TA algorithms presented in [10,11,12] are all based on an assumption that the master SINS can be constantly calibrated by the global position system (GPS), which utilizes the satellites as anchors to provide position information [15]. In this assumption, the error of master SINS is much smaller than the error of slave SINS. Then, the master SINS can be approximately considered as error-free. In practice, the position accuracy of GPS is seriously decreased by the multipath effect in high latitude area [16]. Thus, the master SINS cannot be calibrated by GPS and considered as error-free. In this condition, the master SINS is not accurate and the polar TA algorithms present in [10,11,12] cannot be effective. To ensure the navigation accuracy without the calibration of GPS, some other external navigation methods should be introduced to aid the polar TA. In [17], a star sensor is used in an integrated navigation system of marine SINS. Using the inertial attitude matrix from the star sensor as the reference information, the attitude error of SINS which increases with time can be corrected. In the application of initial alignment, a multiplex two-position alignment method with the aid of star sensor is proposed in [18], which can meet the high accuracy requirements. Star sensors are highly accurate attitude sensitive instruments, widely used for attitude determination [19]. In a rapid TA, the attitude measurement of TA filter is the misalignment angle between master and slave SINSs. In the case that the master SINS has low accuracy, the misalignment angle would be inaccurate and the error of misalignment angle would even more than 1°. Because the star sensor can maintain a high attitude accuracy in the polar region [20], the misalignment angle between the star sensor and slave SINS is accurate. Since the velocity measurement of master SINS is inaccurate and the star sensor cannot provide velocity, an attitude matching method is chosen as the matching method of TA. By choosing the misalignment angle between the star sensor and slave SINS as the measurement of attitude matching method, the alignment accuracy can be improved. In fact, the star sensor measurements are directly used in the filter of TA instead of aiding the master SINS. Therefore, star sensors can be used in assisting TA to accomplish the alignment in the polar region.

In this paper, a new polar TA algorithm with the aid of a star sensor and based on AUKF is proposed. The main contribution of this paper is to ensure and improve the accuracy of polar TA in the condition that the master SINS is inaccurate and the information of system model is abnormal. Nonlinear state equations with the compensation of lever-arm effect are firstly derived under the grid frame. In the case that the master SINS is inaccurate, star sensor is used to keep the high alignment accuracy. By choosing the misalignment angle between star sensor and slave SINS as the measurement, nonlinear measurement equations based on attitude matching method are derived. Then, the filter models for polar TA are designed based on the attitude matching method. An AUKF is applied to promote the alignment accuracy when the information of system model is abnormal. Therefore, the algorithm proposed in this paper can effectively ensure and improve the accuracy of polar TA in the harsh polar environment.

## 2. Polar TA Equations in the Grid Frame

Firstly, the relevant frames used in this paper should be defined: scale symbols

m frame—body frame of master SINS;
s frame—body frame of slave SINS;
$s^{\prime }$ frame—calculated body frame of slave SINS;
G frame—grid frame;
i frame—inertial frame;
t frame—geographic frame;
e frame—earth centered earth fixed frame.

In a grid frame, the plane which parallels to the Greenwich plane and passes through the location of ship is the grid plane. And the intersecting line of grid plane and local-level is the grid north axis line. Grid up axis line coincides with geographic up axis line. The line lies in level and perpendiculars to the grid north axis line is the grid east axis line, which constitute the right frame with the grid north axis [11]. In fact, the grid north axis line is always parallel to the Greenwich meridian. In the grid frame, the North Pole is no longer the geographic pole and the meridian convergence can be avoided. Therefore, the grid frame can solve the problems caused by the meridian convergence in the polar region.

### 2.1. State Equations

#### 2.1.1. Velocity Error Equation

In the ideal condition, the velocity differential equations of master and slave SINSs in the grid frame are as follows [9,10]:

(1) $${\dot{V}}_{m}^{G}=C_{m}^{G}f_{m}^{m}-\left(2\omega_{ie}^{G}+\omega_{eG}^{G}\right)\times V_{m}^{G}+g_{m}^{G}$$

(2) $${\dot{V}}_{s}^{G}=C_{s}^{G}f_{s}^{s}-\left(2\omega_{ie}^{G}+\omega_{eG}^{G}\right)\times V_{s}^{G}+g_{s}^{G}$$

where $V_{m}^{G}$, $C_{m}^{G}$, $f_{m}^{m}$ and $g_{m}^{G}$ are the velocity, attitude matrix, specific force and gravity of master SINS, respectively; $V_{s}^{G}$, $C_{s}^{G}$, $f_{s}^{s}$ and $g_{s}^{G}$ are the velocity, attitude matrix, specific force and gravity of slave SINS in the ideal condition, respectively; $\omega_{ie}^{G}$ is the rotational angular velocity of the earth and $\omega_{eG}^{G}$ is the angular velocity of G frame relative to e frame, which can be expressed as:

(3) $$\omega_{ie}^{G}=C_{g}^{G}\omega_{ie}^{g}=\left[\begin{array}{c} \omega_{iex}^{G}\\ \omega_{iey}^{G}\\ \omega_{iez}^{G} \end{array}\right]=\left[\begin{array}{c} -\omega_{ie}\cos L\sin\sigma \\ \omega_{ie}\cos L\cos\sigma \\ \omega_{ie}\sin L \end{array}\right]$$

(4) $$\omega_{eG}^{G}=\left[\begin{array}{c} \omega_{eGx}^{G}\\ \omega_{eGy}^{G}\\ \omega_{eGz}^{G} \end{array}\right]=\left[\begin{array}{cc} \frac{1}{\tau_{f}} & -\frac{1}{R_{y}}\\ \frac{1}{R_{x}} & -\frac{1}{\tau_{f}}\\ \frac{\kappa }{\tau_{f}} & -\frac{\kappa }{R_{y}} \end{array}\right]\left[\begin{array}{c} V_{G_{E}}\\ V_{G_{N}} \end{array}\right]$$

where $v_{G_{E}}$ and $v_{G_{N}}$ are the east and north velocity in the grid frame, respectively; $R_{x}$, $R_{y}$, $\tau_{f}$ and κ are the simplified calculation parameters which can be expressed as follows:

(5) $$\left\{ \begin{array}{l} \frac{1}{R_{x}}=\frac{\sin^{2}\sigma }{R_{Mh}}+\frac{\cos^{2}\sigma }{R_{Nh}}\\ \frac{1}{R_{y}}=\frac{\cos^{2}\sigma }{R_{Mh}}+\frac{\sin^{2}\sigma }{R_{Nh}}\\ \frac{1}{\tau_{f}}=\left(\frac{1}{R_{Mh}}-\frac{1}{R_{Nh}}\right)\sin\sigma \cos\sigma \\ \kappa =\frac{\sin\lambda \cos L}{\sqrt{1-\cos^{2}L\sin^{2}\lambda }} \end{array}\right.$$

where L and λ is the latitude and longitude, respectively; σ is the angle between true north and grid north axis; $R_{Mh}$ and $R_{Nh}$ are radius of curvature in meridian and prime vertical, respectively.

Due to the errors of IMU and calculation errors in slave SINS, the practical velocity differential equation of slave SINS in the grid frame can be written as:

(6) $${\dot{V}}_{s^{\prime }}^{G}=C_{s^{\prime }}^{G}{\hat{f}}_{s}^{s}-\left(2{\hat{\omega }}_{ie}^{G}+{\hat{\omega }}_{eG}^{G}\right)\times V_{s^{\prime }}^{G}+{\hat{g}}_{s}^{G}$$

where $V_{s^{\prime }}^{G}$ and $C_{s^{\prime }}^{G}$ are the velocity and attitude matrix of slave SINS in the practical condition, respectively; ${\hat{f}}_{s}^{s}$, ${\hat{\omega }}_{ie}^{G}$, ${\hat{\omega }}_{eG}^{G}$ and ${\hat{g}}_{s}^{G}$ are measurement values of slave SINS, respectively; measurement errors $\delta {\hat{f}}_{s}^{s}$, $\delta {\hat{\omega }}_{ie}^{G}$, $\delta {\hat{\omega }}_{eG}^{G}$ and $\delta {\hat{g}}_{s}^{G}$ can be expressed as $\delta {\hat{f}}_{s}^{s}={\hat{f}}_{s}^{s}-f_{s}^{s}$, $\delta {\hat{\omega }}_{ie}^{G}={\hat{\omega }}_{ie}^{G}-\omega_{ie}^{G}$, $\delta {\hat{\omega }}_{eG}^{G}={\hat{\omega }}_{eG}^{G}-\omega_{eG}^{G}$ and $\delta {\hat{g}}_{s}^{G}={\hat{g}}_{s}^{G}-g_{s}^{G}$, respectively, and:

(7) $$\delta \omega_{ie}^{G}=\omega_{ie}^{G}\left[\begin{array}{c} -\cos\varphi \cos\sigma \\ -\cos\varphi \sin\sigma \\ 0 \end{array}\right]\delta \sigma +\left[\begin{array}{c} \sin\varphi \sin\sigma \\ -\sin\varphi \cos\sigma \\ \cos\varphi \end{array}\right]\delta \varphi$$

(8) $$\delta \omega_{eG}^{G}=\left[\begin{array}{ccc} 0 & 0 & \frac{v_{G_{E}}}{R_{e}^{2}}\\ 0 & 0 & -\frac{v_{G_{E}}}{R_{e}^{2}}\\ \frac{v_{G_{N}}\sin\sigma }{R_{e}\sin^{2}\varphi } & 0 & \frac{v_{G_{N}}\cot\varphi \sin\sigma }{R_{e}^{2}} \end{array}\right]\left[\begin{array}{c} \delta \varphi \\ \delta \lambda \\ \delta h \end{array}\right]+\left[\begin{array}{ccc} 0 & -\frac{1}{R_{e}} & 0\\ \frac{1}{R_{e}} & 0 & 0\\ 0 & \frac{-\cot\varphi \sin\sigma }{R_{e}} & 0 \end{array}\right]\left[\begin{array}{c} \delta v_{G_{E}}\\ \delta v_{G_{N}}\\ \delta v_{G_{U}} \end{array}\right]+\left[\begin{array}{c} 0\\ 0\\ \frac{v_{G_{N}}\cot\varphi \sin\sigma }{R_{e}^{2}} \end{array}\right]\delta \sigma$$

where:

(9) $$\delta \sigma =\frac{\sin\varphi }{1-\cos^{2}\varphi \sin^{2}\lambda }\delta \lambda +\frac{\sin\lambda \cos\lambda \cos\varphi }{1-\cos^{2}\varphi \sin^{2}\lambda }\delta \varphi$$

From Equations (7)–(9), we can obtain that $\delta {\hat{\omega }}_{ie}^{G}$ and $\delta {\hat{\omega }}_{eG}^{G}$ are mainly caused by the position errors. During the very short time of TA, the position errors are close to zero. Therefore, $\delta {\hat{\omega }}_{ie}^{G}$ and $\delta {\hat{\omega }}_{eG}^{G}$ can be omitted in TA.

Because:

(10) $$f_{s}^{s}=f_{m}^{s}+a_{r}^{s}=C_{m}^{s}f_{m}^{m}+a_{r}^{s}$$

where $a_{r}^{s}$ is the lever-arm acceleration in ${}_{s}$ frame.

Therefore:

(11) $${\hat{f}}_{s}^{s}=f_{s}^{s}+\delta f_{s}^{s}=C_{m}^{s}f_{m}^{m}+a_{r}^{s}+\delta f_{s}^{s}$$

where $\delta f_{s}^{s}$ is the accelerometer error of slave SINS.

Subtracting Equation (6) from (1):

(12) $$\begin{array}{ll} {\dot{V}}_{s^{\prime }}^{G}-{\dot{V}}_{m}^{G} & =C_{s^{\prime }}^{G}{\hat{f}}_{s}^{s}-C_{m}^{G}f_{m}^{m}-\left(2{\hat{\omega }}_{ie}^{G}+{\hat{\omega }}_{eG}^{G}\right)\times V_{s^{\prime }}^{G}+\left(2\omega_{ie}^{G}+\omega_{eG}^{G}\right)\times V_{m}^{G}+{\hat{g}}_{s}^{G}-g_{m}^{G}\\ & =C_{s^{\prime }}^{G}{\hat{f}}_{s}^{s}-C_{m}^{G}C_{s}^{m}\left({\hat{f}}_{s}^{s}-a_{r}^{s}-\delta f_{s}^{s}\right)-\left(2\omega_{ie}^{G}+\omega_{eG}^{G}\right)\times \left(V_{s^{\prime }}^{G}-V_{m}^{G}\right)+\delta g_{s}^{G}\\ & =C_{s^{\prime }}^{G}\left(I-C_{m}^{s^{\prime }}C_{s}^{m}\right){\hat{f}}_{s}^{s}+C_{m}^{G}a_{r}^{m}+C_{m}^{G}C_{s}^{m}\delta f_{s}^{s}-\left(2\omega_{ie}^{G}+\omega_{eG}^{G}\right)\times \left(V_{s^{\prime }}^{G}-V_{m}^{G}\right)+\delta g_{s}^{G} \end{array}$$

Due to the different locations of master and slave SINSs, the lever-arm effect exists in TA, which produces the lever-arm velocity:

(13) $$V_{r}^{G}=C_{m}^{G}\left(\omega_{im}^{m}\times r^{m}\right)$$

and:

(14) $${\dot{V}}_{r}^{G}=\frac{d}{\mathrm{dt}}\left\{C_{m}^{G}\left(\omega_{im}^{m}\times r^{m}\right)\right\}=\frac{d}{\mathrm{dt}}\left\{C_{i}^{G}C_{m}^{i}\left(\omega_{im}^{m}\times r^{m}\right)\right\}=C_{m}^{G}a_{r}^{m}-\left(\omega_{ie}^{G}+\omega_{eG}^{G}\right)\times V_{r}^{G}$$

Subtracting Equation (12) from (14):

(15) $$\begin{array}{ll} {\dot{V}}_{s^{\prime }}^{G}-{\dot{V}}_{m}^{G}-{\dot{V}}_{r}^{G} & =C_{s^{\prime }}^{G}\left(I-C_{m}^{s^{\prime }}C_{s}^{m}\right){\hat{f}}_{s}^{s}+C_{m}^{G}a_{r}^{m}+C_{m}^{G}C_{s}^{m}\delta f_{s}^{s}-C_{m}^{G}a_{r}^{m}\\ & -\left(2\omega_{ie}^{G}+\omega_{eG}^{G}\right)\times \left(V_{s^{\prime }}^{G}-V_{m}^{G}\right)+\left(\omega_{ie}^{G}+\omega_{eG}^{G}\right)\times V_{r}^{G}+\delta g_{s}^{G}\\ & =C_{s^{\prime }}^{G}\left(I-C_{m}^{s^{\prime }}C_{s}^{m}\right){\hat{f}}_{s}^{s}-\left(2\omega_{ie}^{G}+\omega_{eG}^{G}\right)\times \left(V_{s^{\prime }}^{G}-V_{m}^{G}-V_{r}^{G}\right)+C_{s^{\prime }}^{G}C_{m}^{s^{\prime }}C_{s}^{m}\delta f_{s}^{s}-\omega_{ie}^{G}\times V_{r}^{G} \end{array}$$

Define the velocity error of TA in the grid frame as follows:

(16) $$\Delta V^{G}=V_{s^{\prime }}^{G}-V_{m}^{G}-V_{r}^{G}$$

Thus:

(17) $$\Delta {\dot{V}}^{G}={\dot{V}}_{s^{\prime }}^{G}-{\dot{V}}_{m}^{G}-{\dot{V}}_{r}^{G}$$

and:

(18) $$\Delta {\dot{V}}^{G}=C_{s^{\prime }}^{G}\left(I-C_{m}^{s^{\prime }}C_{s}^{m}\right){\hat{f}}_{s}^{s}-\left(2\omega_{ie}^{G}+\omega_{eG}^{G}\right)\times \Delta V^{G}+C_{s^{\prime }}^{G}C_{m}^{s^{\prime }}C_{s}^{m}\delta f_{s}^{s}-\omega_{ie}^{G}\times V_{r}^{G}$$

Substituting Equation (13) into (18), the velocity equation of polar TA in the grid frame is:

(19) $$\Delta {\dot{V}}^{G}=C_{s^{\prime }}^{G}\left(I-C_{m}^{s^{\prime }}C_{s}^{m}\right){\hat{f}}_{s}^{s}-\left(2\omega_{ie}^{G}+\omega_{eG}^{G}\right)\times \Delta V^{G}+C_{s^{\prime }}^{G}C_{m}^{s^{\prime }}C_{s}^{m}\delta f_{s}^{s}-\omega_{ie}^{G}\times C_{m}^{G}\omega_{im}^{m}\times r^{m}$$

**Figure 1:**
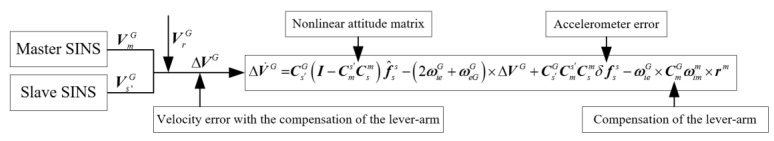
Schematic diagram of the velocity error equation.

In conclusion, the schematic diagram of the velocity error equation is shown in Figure 1:

#### 2.1.2. Attitude Error Equations

In the ideal condition, the attitude differential equations of master and slave SINSs in the grid frame are as follows [9,10]:

(20) $${\dot{C}}_{m}^{G}=C_{m}^{G}\left[\omega_{Gm}^{m}\times \right]$$

(21) $${\dot{C}}_{s}^{G}=C_{s}^{G}\left[\omega_{Gs}^{s}\times \right]$$

where $\omega_{Gs}^{s}$ is the angular velocity of ${}_{s}$ frame relative to G frame and $\omega_{Gm}^{m}$ is the angular velocity of ${}_{m}$ frame relative to G frame, which can be expressed as:

(22) $$\omega_{Gm}^{m}=\omega_{im}^{m}-\omega_{iG}^{m}=\omega_{im}^{m}-C_{G}^{m}\omega_{iG}^{G}$$

where $\omega_{im}^{m}$ is the gyroscope error of master SINS and $\omega_{iG}^{G}$ is the angular velocity of G frame relative to i frame.

Due to the errors of slave SINS, the practical attitude differential equation of slave SINS in the grid frame can be written as:

(23) $${\dot{C}}_{s^{\prime }}^{G}=C_{s^{\prime }}^{G}\left[\omega_{Gs^{\prime }}^{s^{\prime }}\times \right]=C_{s^{\prime }}^{G}\left[{\hat{\omega }}_{Gs}^{s}\times \right]$$

where ${\hat{\omega }}_{Gs}^{s}$ is the practical $\omega_{Gs}^{s}$ measured by slave SINS, which can be presented as:

(24) $${\hat{\omega }}_{Gs}^{s}={\hat{\omega }}_{is}^{s}-{\hat{\omega }}_{iG}^{s}$$

where ${\hat{\omega }}_{iG}^{s}$ is the practical $\omega_{iG}^{G}$ on ${}_{s}$ frame, and ${\hat{\omega }}_{is}^{s}$ is the practical gyroscope output of slave SINS:

(25) $${\hat{\omega }}_{is}^{s}=\omega_{is}^{s}+\delta \omega_{is}^{s}=\omega_{im}^{s}+\omega_{f}^{s}+\delta \omega_{is}^{s}$$

where $\omega_{im}^{s}$ is the gyroscope error of master SINS on ${}_{s}$ frame, $\omega_{f}^{s}$ is the flexural deformation angle and $\delta \omega_{is}^{s}$ is the gyroscope error of slave SINS.

Define the misalignment angle $\phi_{m}^{G}$ between $s^{\prime }$ frame and ${}_{m}$ frame as measurement angle, and it can be described as:

(26) $${\dot{C}}_{s^{\prime }}^{m}=C_{s^{\prime }}^{m}\left[\omega_{ms^{\prime }}^{s^{\prime }}\times \right]=C_{s^{\prime }}^{m}\left[{\hat{\omega }}_{ms}^{s}\times \right]$$

where $C_{s^{\prime }}^{m}$ is the direction cosine matrix from $s^{\prime }$ and ${}_{m}$ frame and ${\hat{\omega }}_{ms}^{s}$ is the practical angular velocity of ${}_{s}$ frame relative to ${}_{m}$ frame.

Refer to using Euler angle algorithm to obtain attitude differential equation in SINS algorithm, $s^{\prime }$ frame can transfer to ${}_{m}$ frame by the rotation of Z axis→X axis→Y axis, and the corresponding rotation angles are $\phi_{mz}^{G}$, $\phi_{mx}^{G}$ and $\phi_{my}^{G}$:

(27) $$\begin{array}{ll} \omega_{ms^{\prime }}^{s^{\prime }} & =\left[\begin{array}{ccc} c\phi_{my}^{G} & 0 & -s\phi_{my}^{G}\\ 0 & 1 & 0\\ s\phi_{my}^{G} & 0 & c\phi_{my}^{G} \end{array}\right]\left[\begin{array}{ccc} 1 & 0 & 0\\ 0 & c\phi_{mx}^{G} & s\phi_{mx}^{G}\\ 0 & -s\phi_{mx}^{G} & c\phi_{mx}^{G} \end{array}\right]\left[\begin{array}{c} 0\\ 0\\ {\dot{\phi }}_{mz}^{G} \end{array}\right]+\left[\begin{array}{ccc} 1 & 0 & 0\\ 0 & c\phi_{mx}^{G} & s\phi_{mx}^{G}\\ 0 & -s\phi_{mx}^{G} & c\phi_{mx}^{G} \end{array}\right]\left[\begin{array}{c} 0\\ {\dot{\phi }}_{my}^{G}\\ 0 \end{array}\right]+\left[\begin{array}{c} {\dot{\phi }}_{mx}^{G}\\ 0\\ 0 \end{array}\right]\\ & =\left[\begin{array}{ccc} c\phi_{my}^{G} & 0 & -c\phi_{mx}^{G}s\phi_{my}^{G}\\ 0 & 1 & s\phi_{mx}^{G}\\ s\phi_{my}^{G} & 0 & c\phi_{mx}^{G}c\phi_{my}^{G} \end{array}\right]\left[\begin{array}{c} {\dot{\phi }}_{mx}^{G}\\ {\dot{\phi }}_{my}^{G}\\ {\dot{\phi }}_{mz}^{G} \end{array}\right]=\left[\begin{array}{ccc} c\phi_{my}^{G} & 0 & -c\phi_{mx}^{G}s\phi_{my}^{G}\\ 0 & 1 & s\phi_{mx}^{G}\\ s\phi_{my}^{G} & 0 & c\phi_{mx}^{G}c\phi_{my}^{G} \end{array}\right]{\dot{\phi }}_{m}^{G} \end{array}$$

where ${}^{c}$ represents ${}^{\cos}$ and ${}^{s}$ represents ${}^{\sin}$.

Thus:

(28) $${\dot{\phi }}_{m}^{G}=C_{\phi }{\hat{\omega }}_{ms}^{s}$$

where:

(29) $$C_{\phi }=\left[\begin{array}{ccc} \cos\phi_{my}^{G} & 0 & \sin\phi_{my}^{G}\\ \tan\phi_{mx}^{G}\sin\phi_{my}^{G} & 1 & \sin\phi_{mx}^{G}\\ \sin\phi_{my}^{G}/\cos\phi_{mx}^{G} & 0 & \cos\phi_{my}^{G}/\cos\phi_{mx}^{G} \end{array}\right]$$

According to Equation (27):

(30) $$C_{m}^{s^{\prime }}=[\begin{array}{c} c\phi_{my}^{G}c\phi_{mz}^{G}-s\phi_{mx}^{G}s\phi_{my}^{G}c\phi_{mz}^{G}\\ -c\phi_{mx}^{G}s\phi_{mz}^{G}\\ s\phi_{my}^{G}c\phi_{mz}^{G}+s\phi_{mx}^{G}c\phi_{my}^{G}s\phi_{mz}^{G} \end{array}\;\begin{array}{cc} c\phi_{my}^{G}s\phi_{mz}^{G}+s\phi_{mx}^{G}s\phi_{my}^{G}c\phi_{mz}^{G} & -c\phi_{mx}^{G}s\phi_{my}^{G}\\ c\phi_{mx}^{G}c\phi_{mz}^{G} & s\phi_{mx}^{G}\\ s\phi_{my}^{G}s\phi_{mz}^{G}-s\phi_{mx}^{G}c\phi_{my}^{G}c\phi_{mz}^{G} & c\phi_{mx}^{G}c\phi_{my}^{G} \end{array}]$$

Similarly, define the misalignment angle $\phi_{a}^{G}$ between $s^{\prime }$ frame and ${}_{m}$ frame as measurement angle, and with the considering of flexural deformation angle θ, it can be described as:

(31) $$C_{m}^{s}=[\begin{array}{c} c\varphi_{y}c\varphi_{z}-s\varphi_{x}s\varphi_{y}c\varphi_{z}\\ -c\varphi_{x}s\varphi_{z}\\ s\varphi_{y}c\varphi_{z}+s\varphi_{x}c\varphi_{y}s\varphi_{z} \end{array}\;\begin{array}{cc} c\varphi_{y}s\varphi_{z}+s\varphi_{x}s\varphi_{y}c\varphi_{z} & -c\varphi_{x}s\varphi_{y}\\ c\varphi_{x}c\varphi_{z} & s\varphi_{x}\\ s\varphi_{y}s\varphi_{z}-s\varphi_{x}c\varphi_{y}c\varphi_{z} & c\varphi_{x}c\varphi_{y} \end{array}]$$

where $\varphi_{i}=\phi_{mi}^{G}+\theta_{i}\;\left(i=x,y,z\right)$.

In the case of large azimuth misalignment angle, $\phi_{mx}^{G}$ and $\phi_{my}^{G}$ are small angle while $\phi_{mz}^{G}$ is large angle, so $C_{\phi }$ becomes $I_{3\times 3}$. Thus:

(32) $${\dot{\phi }}_{m}^{G}={\hat{\omega }}_{ms}^{s}$$

According to Equation (26):

(33) $$\left[{\hat{\omega }}_{ms}^{s}\times \right]=C_{m}^{s^{\prime }}{\dot{C}}_{s^{\prime }}^{m}$$

Because:

(34) $$C_{s^{\prime }}^{m}=C_{G}^{m}C_{s^{\prime }}^{G}$$

The derivative of $C_{s^{\prime }}^{m}$ is:

(35) $${\dot{C}}_{s^{\prime }}^{m}={\dot{C}}_{G}^{m}C_{s^{\prime }}^{G}+C_{G}^{m}{\dot{C}}_{s^{\prime }}^{G}=C_{G}^{m}\left[\omega_{mG}^{G}\times \right]C_{s^{\prime }}^{G}+C_{G}^{m}C_{s^{\prime }}^{G}\left[{\hat{\omega }}_{Gs}^{s}\times \right]$$

Substituting Equation (35) into (33), Equation (33) can be rewritten as:

(36) $$\left[{\hat{\omega }}_{ms}^{s}\times \right]=C_{m}^{s^{\prime }}\left[C_{G}^{m}\left[\omega_{mG}^{G}\times \right]C_{s^{\prime }}^{G}+C_{G}^{m}C_{s^{\prime }}^{G}\left[{\hat{\omega }}_{Gs}^{s}\times \right]\right]=C_{G}^{s^{\prime }}\left[\omega_{mG}^{G}\times \right]C_{s^{\prime }}^{G}+\left[{\hat{\omega }}_{Gs}^{s}\times \right]$$

Because:

(37) $$C_{G}^{s^{\prime }}\left[\omega_{mG}^{G}\times \right]C_{s^{\prime }}^{G}=\left[C_{G}^{s^{\prime }}\omega_{mG}^{G}\times \right]=-\left[\omega_{Gm}^{s^{\prime }}\times \right]$$

Thus:

(38) $$\left[{\hat{\omega }}_{ms}^{s}\times \right]=-\left[\omega_{Gm}^{s^{\prime }}\times \right]+\left[{\hat{\omega }}_{Gs}^{s}\times \right]$$

The vector form of Equation (38) is:

(39) $${\hat{\omega }}_{ms}^{s}=-C_{G}^{s^{\prime }}\omega_{Gm}^{G}+{\hat{\omega }}_{Gs}^{s}=-\omega_{Gm}^{s^{\prime }}+{\hat{\omega }}_{Gs}^{s}=-C_{m}^{s^{\prime }}C_{s}^{m}\omega_{Gm}^{s}+{\hat{\omega }}_{Gs}^{s}$$

therefore:

(40) $${\dot{\phi }}_{m}^{G}=-C_{m}^{s^{\prime }}C_{s}^{m}\omega_{Gm}^{s}+{\hat{\omega }}_{Gs}^{s}$$

where:

(41) $$\omega_{Gm}^{s}=\omega_{im}^{s}-\omega_{iG}^{s}={\hat{\omega }}_{Gs}^{s}-\omega_{fs}^{s}-\delta \omega_{is}^{s}+{\hat{\omega }}_{iG}^{s}-\omega_{iG}^{s}$$

Substituting Equation (42) into (41), the attitude equations of polar TA in the grid frame is:

(42) $$\begin{array}{l} {\dot{\phi }}_{m}^{G}=\left(I-C_{m}^{s^{\prime }}C_{s}^{m}\right){\hat{\omega }}_{Gs}^{s}+C_{m}^{s^{\prime }}C_{s}^{m}\left(\omega_{fs}^{s}+\delta \omega_{is}^{s}-{\hat{\omega }}_{iG}^{s}\right)\\ {\dot{\phi }}_{a}^{G}=0 \end{array}$$

**Figure 2:**
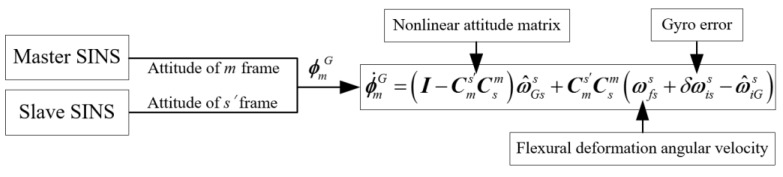
Schematic diagram of the attitude error equation.

In conclusion, the schematic diagram of the attitude error equation is shown in Figure 2:

### 2.2. Measurement Equations

#### 2.2.1. Modeling of the Star Sensor

A star sensor is a charge coupled device (CCD) sensor, which obtains a star atlas using its CCD camera. Through a series of calculations such as extraction of stars, recognition of star atlas and attitude calculation, the attitude of carrier is determined. Star sensors are highly accurate and autonomous sensors, which have an unconstrained field of view and no cumulative error. Thus, star sensors are the most accurate attitude sensitive instruments at present.

Star sensor errors can be modeled as two part errors: measurement error $\omega_{m^{\prime }}$ and installation error δa. As a high-accuracy attitude sensitive instrument, the measurement accuracy of a star sensor can reach arc-second scale. In addition, the measurement error of a star sensor does not accumulate with time. Therefore, the measurement error of a star sensor $\omega_{m^{\prime }}$ can be modeled as a white noise process with zero-mean. Due to the installation error, the $m^{\prime }$ frame does not coincide with ${}_{m}$ frame and the direction cosine matrix from $m^{\prime }$ frame to ${}_{m}$ frame can be expressed as:

(43) $$C_{m^{\prime }}^{m}=\left[\begin{array}{ccc} 1 & \delta a_{z} & -\delta a_{y}\\ -\delta a_{z} & 1 & \delta a_{x}\\ \delta a_{y} & -\delta a_{x} & 1 \end{array}\right]=I-\left(\delta a\times \right)$$

where δa is the installation error of star sensor and can be expressed as follows:

(44) $$\delta a=\delta a_{s}+\delta a_{w}$$

where $\delta a_{s}$ is the constant error and $\delta a_{w}$ is the random error.

The output of star sensor is the direction cosine matrix from $m^{\prime }$ frame to i frame $C_{m^{\prime }}^{i}$, Meanwhile, the direction cosine matrix from G frame to i frame $C_{G}^{i}$ can be obtained real-time. Therefore, the direction cosine matrix from ${}_{m}$ frame to G frame $C_{m^{\prime }}^{G}$ can be calculated as:

(45) $$C_{m^{\prime }}^{G}=C_{i}^{G}C_{m^{\prime }}^{i}$$

#### 2.2.2. Attitude Matching Method

In the case that the master SINS has low accuracy, TA with the aid of external information is necessary to ensure the alignment accuracy. The star sensor can perform well and maintain high accuracy in the polar region, so a star sensor can be used to assist the polar TA.

In a rapid TA, the attitude measurement of TA filter is the misalignment angle between $s^{\prime }$ frame and ${}_{m}$ frame $\phi_{m}^{G}$. However, $\phi_{m}^{G}$ would be inaccurate in the case that the master SINS has low accuracy. In this condition, if $\phi_{m}^{G}$ is still chosen as the attitude misalignment, the accuracy of TA would be decreased. Because the star sensor can maintain a high attitude accuracy in the polar region, the misalignment angle between $s^{\prime }$ frame and $m^{\prime }$ frame $\phi_{s}^{G}$ is more accurate than $\phi_{m}^{G}$. Therefore, $\phi_{s}^{G}$ can be the replacement of $\phi_{m}^{G}$ as the attitude misalignment to improve the accuracy of TA.

In the case of large azimuth misalignment angle, Equation (30) can be rewritten as:

(46) $$C_{m}^{s^{\prime }}=[\begin{array}{c} \cos\phi_{mz}^{G}\\ -\sin\phi_{mz}^{G}\\ \phi_{my}^{G}\cos\phi_{mz}^{G}+\phi_{mx}^{G}\sin\phi_{mz}^{G} \end{array}\;\begin{array}{cc} \sin\phi_{mz}^{G} & -\phi_{my}^{G}\\ \cos\phi_{mz}^{G} & \phi_{mx}^{G}\\ \phi_{my}^{G}\sin\phi_{mz}^{G}-\phi_{mx}^{G}\cos\phi_{mz}^{G} & 1 \end{array}]$$

The direction cosine matrix from $m^{\prime }$ to $s^{\prime }$ frame can be expressed as:

(47) $$C_{m^{\prime }}^{s^{\prime }}=C_{m^{\prime }}^{m}C_{m}^{s^{\prime }}=\left[I-\left(\delta a\times \right)\right]C_{m}^{s^{\prime }}$$

Thus, the measurement vector of attitude matching method can be described as:

(48) $$Z_{att}=\left[\begin{array}{c} C_{m^{\prime }}^{s^{\prime }}\left(2,3\right)\\ C_{m^{\prime }}^{s^{\prime }}\left(1,3\right)\\ C_{m^{\prime }}^{s^{\prime }}\left(1,2\right) \end{array}\right]+\omega_{a}=\left[\begin{array}{c} \delta a_{z}\phi_{my}^{G}+\phi_{mx}^{G}+\delta a_{x}+\omega_{ax}\\ -\phi_{my}^{G}+\delta a_{z}\phi_{mx}^{G}-\delta a_{y}+\omega_{ay}\\ \sin\phi_{mz}^{G}+\delta a_{z}\cos\phi_{mz}^{G}-\delta a_{y}\phi_{my}^{G}\sin\phi_{mz}^{G}+\delta a_{y}\phi_{m\;x}^{G}\cos\phi_{mz}^{G}+\omega_{az} \end{array}\right]$$

where $\omega_{a}={\left[\begin{array}{ccc} \omega_{ax} & \omega_{ay} & \omega_{az} \end{array}\right]}^{T}$ is the white noise of attitude measurement.

Neglecting the small second-order amount, Equation (48) can be rewritten as:

(49) $$Z_{att}=\left[\begin{array}{c} \phi_{mx}^{G}+\delta a_{x}+\omega_{ax}\\ -\phi_{my}^{G}-\delta a_{y}+\omega_{ay}\\ \sin\phi_{mz}^{G}+\delta a_{z}\cos\phi_{mz}^{G}+\omega_{az} \end{array}\right]$$

**Figure 3:**
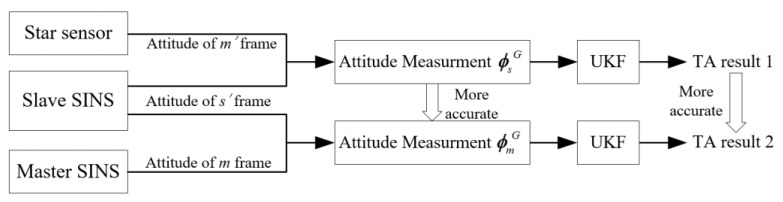
Advantage of TA with the aid of a star sensor.

To explain how the star sensor can help to improve the accuracy, the advantage of TA with the aid of a star sensor is shown in Figure 3:

## 3. Filter Models and Algorithm

Since the measurement information which has relationship with master SINS is inaccurate, this information should be discarded from the measurement. To improve the accuracy of TA, an attitude matching method is chosen as matching method and the accurate $\phi_{s}^{G}$ is used as the measurement. An AUKF is introduced to estimate the states of TA in the case of the harsh polar environment.

### 3.1. Filter Models for Polar TA

Based on the state and measurement equations in chapter 2, the filter models for polar TA, which consists of a dynamic model and an observation model, can be derived in the following subsections. 

At first, the gyro and accelerometer errors of slave SINS can be described as:

(50) $$\delta \omega_{is}^{s}=\varepsilon_{s}^{s}+\varepsilon_{w}^{s};\;\delta f_{s}^{s}=\nabla_{s}^{s}+\nabla_{w}^{s}$$

where $\varepsilon_{s}^{s}$ is the gyro constant drift and $\varepsilon_{w}^{s}$ is the gyro random drift; $\nabla_{s}^{s}$ is the accelerometer constant drift and $\nabla_{w}^{s}$ is the accelerometer random drift.

Then, states of the dynamic model should be defined. The measurement misalignment angle $\phi_{m}^{G}$, actual physical misalignment angle $\phi_{a}^{G}$, velocity error $\Delta V^{G}$, the gyro constant drift $\varepsilon_{s}^{s}$ and accelerometer constant drift $\nabla_{s}^{s}$ are firstly chosen as the states. Then, the lever-arm vector is chosen as the state to estimate and compensate the lever-arm effect. In order to achieve a high accuracy of the attitude measurement, the installation error of star sensor δa is chosen as the state. Therefore, states of the dynamic model are as follows:

(51) $$X={\left[{\left(\Delta V^{G}\right)}^{T}\;{\left(\phi_{m}^{G}\right)}^{T}\;{\left(\varepsilon_{s}^{s}\right)}^{T}\;{\left(\nabla_{s}^{s}\right)}^{T}\;{\left(\phi_{a}^{G}\right)}^{T}\;{\left(r^{m}\right)}^{T}\;{\left(\delta a\right)}^{T}\;\right]}^{T}$$

Combining Equations (19), (42), (44) and (50), the differential equations of states can be described as follows:

(52) $$\{\begin{array}{ll} \Delta {\dot{V}}^{G} & =\;C_{s^{\prime }}^{G}\left(I-C_{m}^{s^{\prime }}C_{s}^{m}\right){\hat{f}}_{s}^{s}-\left(2\omega_{ie}^{G}+\omega_{eG}^{G}\right)\times \Delta V^{G}+C_{s^{\prime }}^{G}C_{m}^{s^{\prime }}C_{s}^{m}\nabla_{s}^{s}-\omega_{ie}^{G}\times C_{m}^{G}\omega_{im}^{m}\times r^{m}\\ {\dot{\phi }}_{m}^{G} & =\left(I-C_{m}^{s^{\prime }}C_{s}^{m}\right){\hat{\omega }}_{Gs}^{s}+C_{m}^{s^{\prime }}C_{s}^{m}\left(\omega_{fs}^{s}+\varepsilon_{s\;}^{s}-{\hat{\omega }}_{iG}^{s}\right)\\ {\dot{\varepsilon }}_{s\;}^{s} & =0\\ {\dot{\nabla }}_{s}^{s} & =0\\ {\dot{\phi }}_{a}^{G} & =0\\ {\dot{r}}^{m} & =0\\ \delta \dot{a} & =0 \end{array}$$

In the case of large azimuth misalignment angle, $\phi_{mx}^{G}$ and $\phi_{my}^{G}$ are small angle while $\phi_{mz}^{G}$ is large angle. By neglecting the small second-order amount, Equations (30) and (31) can be rewritten as:

(53) $$C_{m}^{s^{\prime }}=[\begin{array}{c} c\phi_{mz}^{G}\\ -s\phi_{mz}^{G}\\ \phi_{my}^{G}c\phi_{mz}^{G}+\phi_{mx}^{G}s\phi_{mz}^{G} \end{array}\;\begin{array}{cc} s\phi_{mz}^{G} & -\phi_{my}^{G}\\ c\phi_{mz}^{G} & \phi_{mx}^{G}\\ \phi_{my}^{G}s\phi_{mz}^{G}-\phi_{mx}^{G}c\phi_{mz}^{G} & 1 \end{array}]$$

(54) $$C_{m}^{s}=[\begin{array}{c} c\varphi_{z}\\ -s\varphi_{z}\\ \varphi_{y}c\varphi_{z}+\varphi_{x}s\varphi_{z} \end{array}\;\begin{array}{cc} s\varphi_{z} & -\varphi_{y}\\ c\varphi_{z} & \varphi_{x}\\ \varphi_{y}s\varphi_{z}-\varphi_{x}c\varphi_{z} & 1 \end{array}]$$

Therefore, the system model is nonlinear. In the case of the master SINS is not accurate, $\phi_{s}^{G}$ is the replacement of $\phi_{m}^{G}$ as the attitude misalignment to improve the accuracy of TA. Therefore, attitude matching method is used for filter models of polar TA and the observation can be defined as:

(55) $$Z=\phi_{s}^{G}$$

According to Equation (49), the observation can be expressed as:

(56) $$Z=\left[\begin{array}{c} \phi_{mx}^{G}+\delta a_{x}+\omega_{ax}\\ -\phi_{my}^{G}-\delta a_{y}+\omega_{ay}\\ \sin\phi_{mz}^{G}+\delta a_{z}\cos\phi_{mz}^{G}+\omega_{az} \end{array}\right]$$

### 3.2. Adatipive Unscented Kalman Filter Algorithm

In general, the nonlinear discrete state and measurement equations, such as Equations (52) and (54), can be written as follows:

(57) $$\{\begin{array}{c} x_{k}=f\left(x_{k-1},k-1\right)+q_{k-1}\\ z_{k}=h\left(x_{k},k\right)+r_{k} \end{array}$$

where $x_{k}\in {\mathbb{R}}^{n}$ is the state, $z_{k}\in {\mathbb{R}}^{n}$ is the measurement, $q_{k}∼N\left(0,Q_{k-1}\right)$ is the Gaussian system noise, $r_{k}∼N\left(0,R_{k}\right)$ is the Gaussian measurement noise, f(·) is the nonlinear function of the state and h(·) is the nonlinear function of the measurement.

The algorithm procedure of UKF can be described as follows:

1. Initialization of state parameter:
  (58) $$\{\begin{array}{c} {\hat{x}}_{0}=E\left[x_{0}\right]\\ P_{0}=E\left[\left(x_{0}-{\hat{x}}_{0}\right){\left(x_{0}-{\hat{x}}_{0}\right)}^{T}\right] \end{array}$$
2. Calculation of the sigma points:
  (59) $$\chi_{k-1}=\left[\begin{array}{ccc} {\hat{x}}_{k-1} & {\hat{x}}_{k-1}+{\left(\sqrt{\left(n+\lambda \right)P_{k-1}}\right)}_{i} & {\hat{x}}_{k-1}-{\left(\sqrt{\left(n+\lambda \right)P_{k-1}}\right)}_{i} \end{array}\right],i=1,2,\ldots ,n$$
  and the associated weights:
  (60) $$\left\{ \begin{array}{l} W_{0}^{m}=\lambda /\left(n+\lambda \right)\\ W_{0}^{c}=\lambda /\left(n+\lambda \right)+\left(1-\alpha^{2}+\beta \right)\\ W_{i}^{m}=W_{i}^{c}=0.5/\left(n+\lambda \right),i=1,2,\ldots ,2n \end{array}\right.$$
  where $W_{i}^{m}$ and $W_{i}^{c}$ are the associated weights of mean value and covariance, respectively.
3. Time Updating:
  (61) $$\chi_{i,k|k-1}=f\left(\chi_{i,k-1}\right),{\hat{x}}_{k}^{-}=\sum_{i=0}^{2n}W_{i}^{m}\chi_{i,k|k-1}$$
  (62) $$P_{k}^{-}=\sum_{i=0}^{2n}W_{i}^{c}\left[\chi_{i,k|k-1}-{\hat{x}}_{k}^{-}\right]{\left[\chi_{i,k|k-1}-{\hat{x}}_{k}^{-}\right]}^{T}+Q_{k}$$
  (63) $$z_{i,k|k-1}=h\left(\chi_{i,k-1}\right),{\hat{z}}_{k}^{-}=\sum_{i=0}^{2n}W_{i}^{m}z_{i,k|k-1}$$
4. Measurement Updating
  (64) $$P_{z_{k},z_{k}}=\sum_{i=0}^{2n}W_{i}^{c}\left[z_{i,k|k-1}-{\hat{z}}_{k}^{-}\right]{\left[z_{i,k|k-1}-{\hat{z}}_{k}^{-}\right]}^{T}+R_{k}$$
  (65) $$P_{x_{k},z_{k}}=\sum_{i=0}^{2n}W_{i}^{c}\left[\chi_{i,k|k-1}-{\hat{x}}_{k}^{-}\right]{\left[z_{i,k|k-1}-{\hat{z}}_{k}^{-}\right]}^{T}$$
  (66) $$K_{k}=P_{x_{k},z_{k}}\cdot P_{z_{k},x_{k}}^{-1},{\hat{x}}_{k}={\hat{x}}_{k}^{-}+K_{k}\left(z_{k}-{\hat{z}}_{k}^{-}\right)$$
  (67) $$P_{k}=P_{k}^{-}-K_{k}\cdot P_{z_{k},z_{k}}\cdot K_{k}^{T}$$

Although UKF is effective in estimating the nonlinear states, it still has some limitations. Firstly, UKF is sensitive to the initial value choice. If the initial value has an error, the performance of the UKF would be reduced. Secondly, the interference of environment and unconfirmed statistical characteristic of noise would also decrease the accuracy of UKF. Therefore, an AUKF is proposed to estimate the misalignment angles of TA in the harsh polar environment.

The algorithm procedure of AUKF is similar to that of UKF. The differences of AUKF are Equations (64), (66) and (67):

(68) $$P_{z_{k},z_{k}}=\frac{1}{\alpha_{k}}\sum_{i=0}^{2n}W_{i}^{c}\left[z_{i,k|k-1}-{\hat{z}}_{k}^{-}\right]{\left[z_{i,k|k-1}-{\hat{z}}_{k}^{-}\right]}^{T}+R_{k}$$

(69) $$P_{x_{k},z_{k}}=\frac{1}{\alpha_{k}}\sum_{i=0}^{2n}W_{i}^{c}\left[\chi_{i,k|k-1}-{\hat{x}}_{k}^{-}\right]{\left[z_{i,k|k-1}-{\hat{z}}_{k}^{-}\right]}^{T}$$

(70) $$P_{k}=\frac{1}{\alpha_{k}}P_{k}^{-}-K_{k}\cdot P_{z_{k},z_{k}}\cdot K_{k}^{T}$$

where $\alpha_{k}$ is the adaptive factor which satisfies $0\leq \alpha_{k}\leq 1$. With the proper value of $\alpha_{k}$, the prediction and measurement information of system model can be balanced. The calculation of $\alpha_{k}$ is as follows:

(71) $$\alpha_{k}=\left\{ \begin{array}{ll} 1, & tr(v_{k}v_{k}{}^{T})\leq tr(P)\\ \frac{tr(P)}{tr(v_{k}v_{k}{}^{T})}, & tr(v_{k}v_{k}{}^{T})>tr(P) \end{array}\right.$$

where $v_{k}$ is the residual error and $v_{k}=z_{k}-{\hat{z}}_{k}^{-}$.

From Equation (69) we can obtain that when the information of system model becomes abnormal, $\alpha_{k}$ would gradually toward to zero, which means that the information of system model is abandoned. It is obviously that the adaptive factor $\alpha_{k}$ can adaptively adjust the state information by using measurement information and residual error.

## 4. Results

With the aid of a star sensor and based on the AUKF, a new polar TA algorithm is proposed in this paper. In order to test and verify the effect of the new polar TA algorithm, an experiment is conduct in this chapter, which also includes the results and analyses.

Because the authors’ country is located in the mid-low latitude region, it is hard to conduct experiments in the polar region. To solve the problem caused by geographic restriction, the experiment is conducted in the form of semi-physical simulation.

### 4.1. Experiment Condition

In practical application, the experimental data of Inertial Measurement Unit (IMU) can be expressed as follows:

(72) $$\{\begin{array}{c} {\hat{\omega }}_{ib}^{b}=\omega_{ib}^{b}+\delta \omega_{ib}^{b}\\ {\hat{f}}^{b}=f^{b}+\delta f^{b} \end{array}$$

where the superscript b represents the body frame of SINS, which includes master and slave body frames—${}_{m}$ frame and ${}_{s}$ frame. Thus, ${\hat{\omega }}_{ib}^{b}$ is the practical angular velocity measured by gyro, $\delta \omega_{ib}^{b}$ is the gyro drifts and $\omega_{ib}^{b}$ is the true angular velocity of SINS. So is the data of accelerometer.

**Figure 4:**
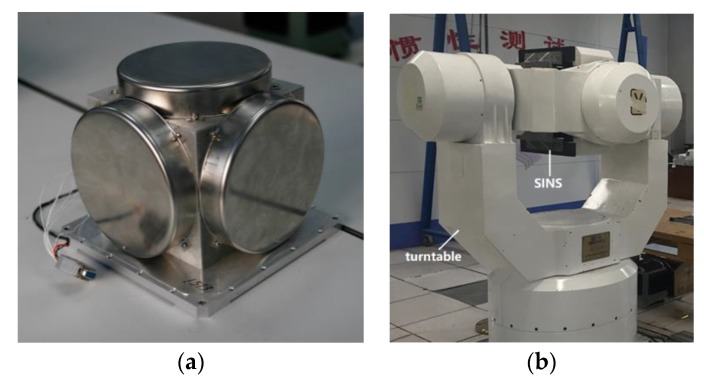
Experimental tools. (**a**) IMU; (**b**) SINS and turntable.

No matter the data is gained from simulation or experiment, the true values of IMU $\omega_{ib}^{b}$ and $f^{b}$ are same. Once the attitude variation and maneuvers of the ship are confirmed, the values of $\omega_{ib}^{b}$ and $f^{b}$ can be gained by simulation. Moreover, the drifts of IMU $\delta \omega_{ib}^{b}$ and $\delta f^{b}$ can be extracted from practical measured data, which is provided by the experimental tools shown in Figure 4.

As shown in Figure 4, the IMU, which consists of three-axis gyroscopes and accelerometers, is contained in a SINS. Meanwhile, the SINS and IMU are installed on a turntable, which can provide a high-precision three-axis rotary movement. Through the test in non-polar areas, the practical measured data which contains IMU biases can be provided. Thus, the IMU data of experiment can be obtained from the practical measured data and simulation data in non-polar areas.

In order to make the experiment closely to the practical application, various sea states and ship maneuvers are considered. The sea states include calm and medium sea states, and the maneuvers include static, uniform linear motion and linear motion with constant acceleration.

(1) In this paper, attitudes of ship are set as sine functions and are set as follows: when ship sails in calm sea state, the amplitude/period of pitch angle, roll angle and yaw angle are 1°/3 s, 1°/5 s and 1°/7 s, respectively; when ship sails in medium sea state, the amplitude/period of pitch angle, roll angle and yaw angle are 9°/3 s, 6°/5 s and 8°/7 s, respectively; the initial phase and heading are 0° and 0°, respectively. units not in italics and with a space after number
(2) Maneuvers of ship are set as follows: the initial latitude φ is 85° and initial longitude λ is 130°; when ship is in uniform linear motion, the velocity of ship is set as 10 nm/h; when ship is in linear motion with constant acceleration, the initial velocity of ship is set as 10 nm/h and the acceleration of ship is set as 0.1 m/s^2^.
(3) Drifts of IMU extracted from practical measured data are as follows: the three-axis gyro constant drifts are $-5.4217\times 10^{-9}\;$ rad/s, $6.9875\times 10^{-9}\;$ rad/s and $2.0264\times 10^{-8}\;$ rad/s, respectively; the three-axis accelerometer constant drifts are $-4.3785\times 10^{-6}\;$ m/s^2^, $5.1478\times 10^{-6}\;$ m/s^2^ and $4.6584\times 10^{-6}$ m/s^2^, respectively; the three-axis gyro random drifts variances are ${\left(9.785\times 10^{-7}\;\mathrm{rad}/s\right)}^{2}$, ${\left(4.527\times 10^{-6}\;\mathrm{rad}/s\right)}^{2}$ and ${\left(2.874\times 10^{-6}\;\mathrm{rad}/s\right)}^{2}$, respectively; the three-axis accelerometer random drifts variances are ${\left(0.00245\;{m/s}^{2}\right)}^{2}$, $\;{\left(0.00578\;{m/s}^{2}\right)}^{2}$ and ${\left(0.000624\;{m/s}^{2}\right)}^{2}$, respectively.
(4) In the case of a large azimuth misalignment angle, the true values of actual physical misalignment angles are set as $\phi_{ax}^{G}=0.6{}^{\circ}$, $\phi_{ay}^{G}=0.4{}^{\circ}$ and $\phi_{ax}^{G}=8{}^{\circ}$, respectively. The lengths of three-axis lever-arm $r^{m}$ are 5m, 0m and 2m, respectively. The constant installation error of star sensor $\delta a_{s}$ are set as 0.08°, 0.07° and 0.09°, respectively; the random installation error of star sensor $\delta a_{w}$ are set as 0.02°, 0.03° and 0.05°, respectively. Simulation time is 60 s and filter frequency is 100 Hz. In the condition that the master SINS is not accurate, the initial attitude errors of master SINS are set as 1.2°, 1.5° and 2.8°, respectively.
(5) The initial state estimation covariance matrix $P_{0}$, system process noise covariance matrix Q, and measurement noise covariance matrix R are set as follows:
  (73) $$\begin{array}{l} P_{011}=P_{022}=P_{033}={\left(0.1\;m/s\right)}^{2},P_{044}={\left(1.2{}^{\circ}\right)}^{2},P_{055}={\left(1.5{}^{\circ}\right)}^{2},P_{066}={\left(2.8{}^{\circ}\right)}^{2}\\ P_{077}=\;{\left(5.4217\times 10^{-9}\;\mathrm{rad}/s\right)}^{2},P_{088}=\;{\left(6.9875\times 10^{-9}\;\mathrm{rad}/s\right)}^{2},P_{099}=\;{\left(2.0264\times 10^{-8}\;\mathrm{rad}/s\right)}^{2}\\ P_{01010}=\;{\left(4.3785\times 10^{-6}\;{m/s}^{2}\;\right)}^{2}\;,P_{01111}=\;{\left(5.1478\times 10^{-6}\;{m/s}^{2}\right)}^{2},P_{01212}=\;{\left(4.6584\times 10^{-6}\;{m/s}^{2}\right)}^{2}\\ P_{01313}={\left(0.6{}^{\circ}\right)}^{2},P_{01414}={\left(0.4{}^{\circ}\right)}^{2},P_{01515}={\left(8{}^{\circ}\right)}^{2},P_{01616}={\left(5\;m\right)}^{2},P_{01717}=0,P_{01818}={\left(2\;m\right)}^{2}\\ P_{01919}={\left(0.08{}^{\circ}\right)}^{2},P_{02020}={\left(0.07{}^{\circ}\right)}^{2},P_{02121}={\left(0.09{}^{\circ}\right)}^{2}\\ Q_{11}={\left(9.785\times 10^{-7}\;\mathrm{rad}/s\right)}^{2}\;,Q_{22}={\left(4.527\times 10^{-6}\;\mathrm{rad}/s\right)}^{2},Q_{33}={\left(2.874\times 10^{-6}\;\mathrm{rad}/s\right)}^{2}\\ Q_{44}={\left(0.00245\;{m/s}^{2}\right)}^{2},Q_{55}=\;{\left(0.00578\;{m/s}^{2}\right)}^{2},Q_{66}={\left(0.000624\;{m/s}^{2}\right)}^{2}\\ Q_{77}={\left(0.02{}^{\circ}\right)}^{2},Q_{88}={\left(0.03{}^{\circ}\right)}^{2},Q_{99}={\left(0.05{}^{\circ}\right)}^{2}\\ R_{11}=R_{22}=R_{33}={\left(0.1\;m/s\right)}^{2},R_{44}=R_{55}=R_{66}={\left(0.01\pi /180\;\mathrm{rad}\right)}^{2} \end{array}$$
(6) The initializations of the filter state vector are set as follows:
  (74) $$\begin{array}{l} X_{1}=X_{2}=X_{3}=0.1\;m/s,X_{4}=1.2{}^{\circ},X_{5}=1.5{}^{\circ},X_{6}=2.8{}^{\circ}\\ X_{7}=5.4217\times 10^{-9}\;\mathrm{rad}/s,X_{8}=6.9875\times 10^{-9}\;\mathrm{rad}/s,X_{9}=2.0264\times 10^{-8}\;\mathrm{rad}/s\\ X_{10}=4.3785\times 10^{-6}\;{m/s}^{2}\;,X_{11}=5.1478\times 10^{-6}\;{m/s}^{2},X_{12}=4.6584\times 10^{-6}\;{m/s}^{2}\\ X_{13}=0.6{}^{\circ},X_{14}=0.4{}^{\circ},X_{15}=8{}^{\circ},X_{16}=5\;m,X_{17}=0,X_{18}=2\;m\\ X_{19}=0.08{}^{\circ},X_{20}=0.07{}^{\circ},X_{21}=0.09{}^{\circ} \end{array}$$

### 4.2. Results and Analyses

**Figure 5:**
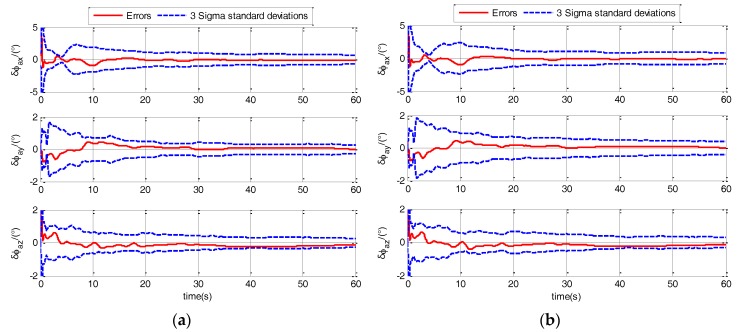
Comparison of 3-sigma standard deviations with the errors when ship sails in calm sea static. (**a**) when ship is static; (**b**) when ship sails in uniform linear motion.

**Figure 6:**
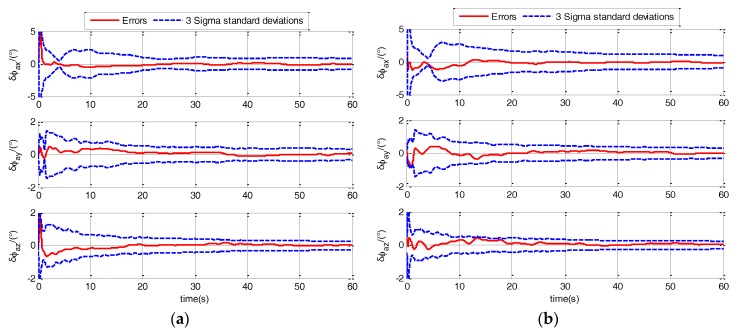
Comparison of 3-sigma standard deviations with the errors when ship sails in medium sea static. (**a**) when ship sails in uniform linear motion; (**b**) when ship sails in linear motion with constant acceleration.

In the rapid TA, the estimation errors of actual misalignment angle $\phi_{a}^{G}$ is the main parameter to evaluate the performance of TA. The 3-sigma standard deviations (3σ) estimated by the filter is a parameter to evaluate the performance of filter. Firstly, a comparison should be conducted to check that the initialization was correct and whether the filter performs consistently. The comparison of 3-Sigma standard deviations with the errors are shown in Figure 5 and Figure 6.

As shown in Figure 5 and Figure 6, the errors are less than +3σ and greater than −3σ under different sea states and maneuvers. The errors are within the range of 3-sigma standard deviations (±3σ), which indicates that the filter is correctly tuned and the initialization was correct. Then, the verifications for the superiority of designed TA model and the performance of AUKF are conducted in the following sections.

#### 4.2.1. Verification for the Superiority of Designed TA Model

With the aid of star sensor and based on the AUKF, a new polar TA algorithm proposed in this paper aims to improve the accuracy of polar TA when master SINS is not accurate. In order to verify the performance of the new polar TA algorithm, it is necessary to introduce the comparison validation. Firstly, the superiority of the designed TA model should be verified. Thus, the new polar TA model proposed in this paper is defined Model 1. The previous polar TA model proposed in [11] is defined as Model 2, which is described as:

(75) $$\{\begin{array}{l} \delta {\dot{V}}^{G}=\left(C_{s^{\prime }}^{G}-C_{s}^{G}\right)f^{s}-\left(2\omega_{ie}^{G}+\omega_{eG}^{G}\right)\times \delta V_{s^{\prime }}^{G}\;-\left(2\delta \omega_{ie}^{G}+\delta \omega_{eG}^{G}\right)\times V^{G}+C_{s^{\prime }}^{G}\delta f+C_{s^{\prime }}^{G}\nabla_{s}^{s}\\ {\dot{\phi }}_{m}^{G}=\left(C_{m}^{s}-C_{m}^{s^{\prime }}\right)\omega_{Gm}^{m}+\omega_{f}-\varepsilon_{s}^{s}-\varepsilon_{w}^{s}\\ \begin{array}{l} {\dot{\nabla }}_{s}^{s}=0\\ {\dot{\varepsilon }}_{s}^{s}=0 \end{array}\\ {\dot{\phi }}_{a}^{G}=0 \end{array}$$

(76) $$Z={\left[\begin{array}{cccccc} \delta V_{x}^{G} & \delta V_{x}^{G} & \delta V_{x}^{G} & \phi_{mx}^{G} & \phi_{my}^{G} & \phi_{mx}^{G} \end{array}\right]}^{T}$$

**Figure 7:**
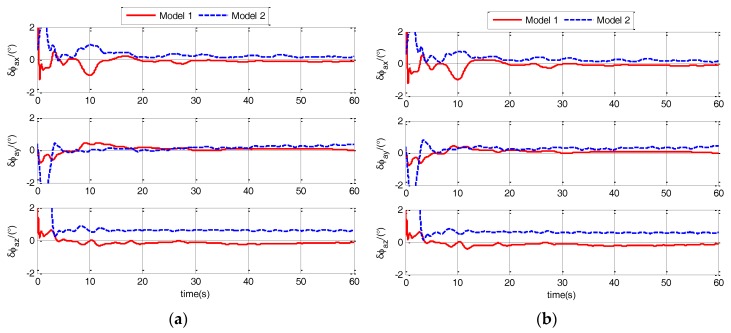
Estimation errors of $\phi_{a}^{G}$ when ship sails in calm sea static for different models. (**a**) when ship is static; (**b**) when ship sails in uniform linear motion.

**Figure 8:**
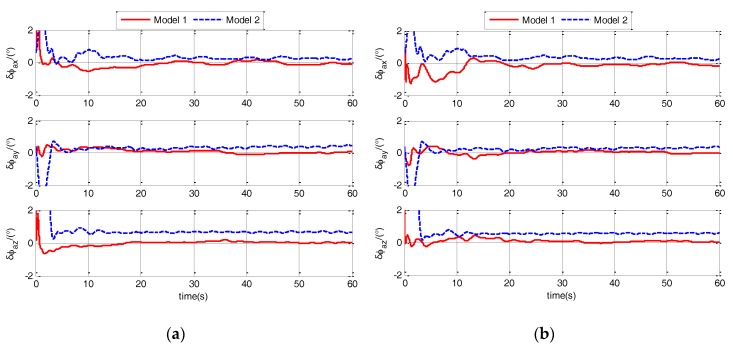
Estimation errors of $\phi_{a}^{G}$ when ship sails in medium sea static for different models. (**a**) when ship sails in uniform linear motion; (**b**) when ship sails in linear motion with constant acceleration.

In calm and medium sea state, the estimation errors of $\phi_{a}^{G}$ for different models are shown in Figure 7 and Figure 8.

Estimation errors of $\phi_{a}^{G}$ under different sea states and maneuvers are shown in Figure 7 and Figure 8. From the figures, it is obvious that the estimation errors of Model 1 are less than the errors of Model 2. This shows that the Model 1 has more improvement in the accuracy of polar TA than Model 2. To detailed analysis the improvement effect, estimation errors (RMS) of $\phi_{a}^{G}$ for different models are shown in Table 1.

As shown in Table 1, three-axis estimation errors of Model 1 are less than the errors of Model 2. Compared with Model 2, the $\phi_{ax}^{G}$, $\phi_{ay}^{G}$ and $\phi_{az}^{G}$ errors of Model 1 are all decreased and the decreased amounts are as follows:

(1) $\phi_{ax}^{G}$: when ship is in calm sea state, the static and ULM errors of Model 1 are decreased to 42.0% and 42.6%, respectively; when ship is in medium sea state, the ULM and LMAC errors of Model 1 are decreased to 38.4% and 36.6%, respectively.
(2) $\phi_{ay}^{G}$: when ship is in calm sea state, the static and ULM errors of Model 1 are decreased to 29.8% and 22.4%, respectively; when ship is in medium sea state, the ULM and LMAC errors of Model 1 are decreased to 20.1% and 28.2%, respectively.
(3) $\phi_{az}^{G}$: when ship is in calm sea state, the static and ULM errors of Model 1 are decreased to 30.23% and 31.97%, respectively; when ship is in medium sea state, the ULM and LMAC errors of Model 1 are decreased to 10.8% and 11.7%, respectively.

Results of Models 1 and 2 demonstrate that Model 1 is obviously superior to Model 2 in promoting the accuracy of polar TA in the case of the master SINS is not accurate.

#### 4.2.2. Verification for the Performance of AUKF

**Figure 9:**
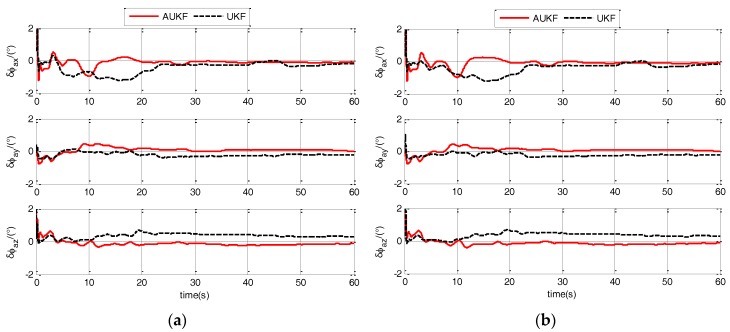
Estimation errors of $\phi_{a}^{G}$ when ship sails in calm sea static of different filters. (**a**) when ship is static; (**b**) when ship sails in uniform linear motion.

**Figure 10:**
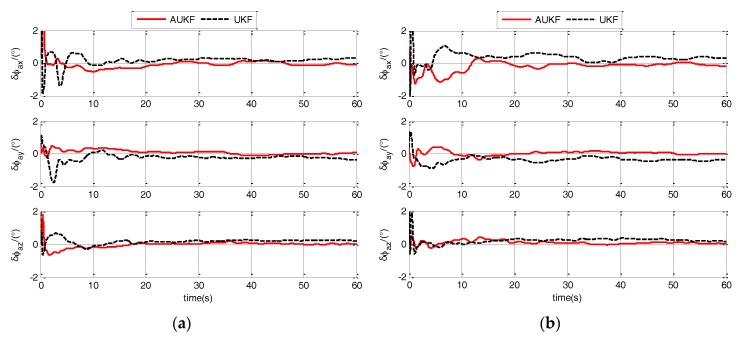
Estimation errors of $\phi_{a}^{G}$ when ship sails in medium sea static of different filters. (**a**) when ship sails in uniform linear motion; (**b**) when ship sails in linear motion with constant acceleration.

Because the algorithm proposed in this paper is based on the AUKF, a compared algorithm should be introduced to verify the effectiveness of the AUKF. Therefore, the second compared algorithm has the same model with the algorithm proposed in this paper but it is based on an UKF. In calm and medium sea state, the estimation errors of $\phi_{a}^{G}$ for different models are shown in Figure 9 and Figure 10.

As shown in Figure 9 and Figure 10, the errors of AUKF are less than the errors of UKF. And it indicates that AUKF is more adaptive than UKF in responding to the unconfirmed statistical characteristic of noise caused by the harsh polar environment. Estimation errors (RMS) of $\phi_{a}^{G}$ for different models are shown in Table 2 to detailed analysis the improvement effect.

As shown in Table 2, three-axis estimation errors of AUKF are less than the errors of UKF. Compared with UKF, the $\phi_{ax}^{G}$, $\phi_{ay}^{G}$ and $\phi_{az}^{G}$ errors of AUKF are all decreased and the decreased amounts are as follows:

(1) $\phi_{ax}^{G}$: when ship is in calm sea state, the static and ULM errors of Model 1 are decreased to 37.5% and 37.1%, respectively; when ship is in medium sea state, the ULM and LMAC errors of Model 1 are decreased to 44.3% and 37.2%, respectively.
(2) $\phi_{ay}^{G}$: when ship is in calm sea state, the static and ULM errors of Model 1 are decreased to 28.7% and 28.9%, respectively; when ship is in medium sea state, the ULM and LMAC errors of Model 1 are decreased to 31.5% and 24.9%, respectively.
(3) $\phi_{az}^{G}$: when ship is in calm sea state, the static and ULM errors of Model 1 are decreased to 50.9% and 51.2%, respectively; when ship is in medium sea state, the ULM and LMAC errors of Model 1 are decreased to 31.3% and 24.3%, respectively.

Results of AUKF and UKF indicate that AUKF has more effectiveness than UKF to ensure and improve the TA performance in responding to the harsh environment of the polar region.

## 5. Discussion

As shown in the results, the new polar TA algorithm with the aid of a star sensor and based on AUKF is more effective than the state-of-the-art algorithms. Compared with the state-of-the-art algorithms, the new polar TA algorithm proposed in this paper has the following advantages:

(1) In the condition that the master SINS has low accuracy, the measurement information would be inaccurate. In Model 2, the measurement $V^{G}$ and $\phi_{m}^{G}$ become inaccurate due to the low accurate master SINS, so the alignment accuracy of Model 2 is decreased. Because the star sensor can keep a high attitude accuracy in the polar region, the misalignment angle between $s^{\prime }$ frame and $m^{\prime }$ frame $\phi_{s}^{G}$ is more accurate than $\phi_{m}^{G}$. By choosing attitude matching method and using $\phi_{s}^{G}$ as the measurement, measurement information of Model 1 can keep accurate. Meanwhile, due to the estimate of δa, the value of $\phi_{s}^{G}$ can be constant corrected. Thus, Model 1 can achieve a high alignment accuracy with the aid of star sensor.
(2) Model 2 does not consider and compensate the lever arm effect, which exists in the practical application of TA. By estimating and compensating the lever-arm $r^{m}$, Model 1 can reduce and eliminate the influence caused by lever-arm. Therefore, Model 1 has more effectiveness in promoting the accuracy of TA.
(3) Since the harsh environment of the polar region, the information of system model is abnormal. With the proper value of adaptive factor, AUKF can balance the prediction and measurement information and adaptively adjust the state information. Thus, the proposed algorithm based on AUKF can and perform better than the state-of-the-art algorithm based on UKF in adjusting the harsh polar environment and improving the estimating accuracy.

Due to the aid of a star sensor and the compensation of lever-arm effect, Model 1 has a higher accuracy than Model 2. Moreover, the performance of AUKF is better than that of UKF in improving the accuracy of polar TA. Therefore, the new polar TA algorithm proposed in this paper is more effective than the state-of-the-art algorithms.

## 6. Conclusions

In the case of the low accurate master SINS and the abnormal system model information caused by the harsh polar environment, a new polar TA algorithm with the aid of a star sensor and based on AUKF is proposed. Nonlinear state equations with the compensation of lever-arm effect are derived in the grid frame. Then, the star sensor is modeled and the accurate $\phi_{s}^{G}$ is chosen to replace the innaccurate $\phi_{m}^{G}$. By choosing attitude matching method and using $\phi_{s}^{G}$ as the measurement, the nonlinear measurement equations are derived. Combined the state and measurement equations, the filter models for polar TA are designed. To solve the abnormal information of system model, an AUKF is introduced to estimate the states of the designed polar TA models. Experimental results have demonstrated that polar TA algorithm proposed in this paper is obviously superior in ensuring and improving the accuracy of polar TA, especially in the harsh environment.

## Figures and Tables

**Table 1:** Estimation errors (RMS) for different models (ULM = Uniform linear motion, LMAC = Linear motion with constant acceleration).

Parameters	Model	Calm Sea State		Medium Sea State	
		Static	ULM	ULM	LMAC
$\phi_{ax}^{G}$/(°)	Model 1	0.0894	0.0920	0.1027	0.1122
	Model 2	0.2128	0.2159	0.2673	0.3066
$\phi_{ay}^{G}$/(°)	Model 1	0.0721	0.0723	0.0753	0.0876
	Model 2	0.2418	0.3233	0.3746	0.3102
$\phi_{az}^{G}$/(°)	Model 1	0.1853	0.1865	0.0717	0.0659
	Model 2	0.6129	0.5834	0.6654	0.5638

**Table 2:** Estimation errors (RMS) of different filters (ULM = Uniform linear motion, LMAC = Linear motion with constant acceleration).

Parameters	Filter	Calm Sea State		Medium Sea State	
		Static	ULM	ULM	LMAC
$\phi_{ax}^{G}$/(°)	AUKF	0.0894	0.0920	0.1027	0.1122
	UKF	0.2384	0.2477	0.2318	0.3015
$\phi_{ay}^{G}$/(°)	AUKF	0.0721	0.0723	0.0753	0.0876
	UKF	0.2515	0.2505	0.2394	0.3513
$\phi_{az}^{G}$/(°)	AUKF	0.1853	0.1865	0.0717	0.0659
	UKF	0.3639	0.3641	0.2292	0.2709
